# Development of a versatile [^68^Ga]Ga-FAPI-46 automated synthesis suitable to multi-elutions of germanium-68/gallium-68 generators

**DOI:** 10.3389/fchem.2024.1411312

**Published:** 2024-07-15

**Authors:** Louis-Paul Paty, Simon Degueldre, Claire Provost, Camille Schmitt, Laura Trump, Julien Fouque, Charles Vriamont, Frank Valla, Thibault Gendron, Olivier Madar

**Affiliations:** ^1^ Département de Radiopharmacologie, Ensemble Hospitalier de l’Institut Curie, Saint-Cloud, France; ^2^ Trasis, Rue Gilles Magnée, Ans, Belgium; ^3^ Centre de Recherche de l’Institut Curie, Laboratoire d’Imagerie Translationnelle en Oncologie (LITO), Orsay, France; ^4^ SOFIE, iTheranostics, Dulles, VA, United States

**Keywords:** PET, FAPI radiopharmaceuticals, radiosynthesis, automation, prepurification, gallium-68

## Abstract

Gallium-68-labeled FAPI-46 has recently been proposed as a novel positron emission tomography imaging probe to diagnose and monitor a wide variety of cancers. Promising results from several ongoing clinical trials have led to a soaring demand for this radiotracer. Typical [^68^Ga]Ga-FAPI-46 labeling protocols do not cope with multiple generator elutions, leaving radiopharmacies unable to scale-up the production and meet the demand. Here, we propose a robust and efficient automated radiosynthesis of [^68^Ga]Ga-FAPI-46 on the Trasis miniAllinOne synthesizer, featuring a prepurification step which allows multiple generator elutions and ensures compatibility with a wide range of gallium-68 generators. Our approach was to optimize the prepurification step by first testing five different cationic cartridge chemistries. Only the strong cationic exchange (SCX) cartridges tested had sufficient affinities for quantitative trapping of >99.9%, while the weak cationics did not exceed 50%. Packaging, rinsing, or flowing of the selected SCX cartridges was not noticeable, but improvements in fluidics managed to save time. Based on our previous development experience of [^68^Ga]Ga-FAPI-46, radiolabeling optimization was also carried out at different temperatures during 10 min. At temperatures above 100°C, radiochemical yield (RCY) > 80% was achieved without significantly increasing the chemical impurities (<5.5 μg mL^-1^). The optimized sequence was reproducibly conducted with three different brands of widely used generators (RCY >88%). A comparison with radiosyntheses carried out without prepurification steps was also conclusive in terms of RCY, radiochemical yield, and chemical purity. Finally, high-activity tests using elutions from three generators were also successful for these parameters. [^68^Ga]Ga-FAPI-46 was consistently obtained in good radiochemical yields (>89%, *n = 3*), and the final product quality was compliant with internal specifications based on European Pharmacopoeia. This process is suitable for GMP production and allows scaling-up of routine productions, higher throughput, and, ultimately, better patient care.

## 1 Introduction

Positron emission tomography (PET) is an essential imaging technique used to diagnose, stage, and assess treatment response. Thanks to unparalleled sensitivity and the continuous development of novel radiotracers, this technique plays an important role in clinical practice and, most notably, in oncology. Cancers are composed of tumor cells and tumor stroma, the latter being a significant part of the tumor and mainly composed of cancer-associated fibroblasts (CAFs) (Fitzgerald and Weiner, 2020). The fibroblast activation protein (FAP) is a transmembrane serine protease (Edosada et al., 2006) expressed by CAF. FAP overexpression is associated with poor cancer prognosis (Park et al., 1999; Brennen et al., 2012; Kratochwil et al., 2019) and is, therefore, an excellent biomarker for cancer and an important target for diagnosis and therapeutic applications in nuclear medicine. The development of fibroblast activation protein inhibitors (FAPIs) radiolabeled with positron-emitting radionuclides led to specific PET radiotracers for the imaging of FAP. Amongst these, gallium-68-labeled FAPI-46 holds great potential for applications in oncology (Mona et al., 2022) or inflammatory diseases (Treglia and Albano, 2023).

Promising results from several ongoing clinical trials with [^68^Ga]Ga-FAPI-46 (Mori et al., 2023) have led to a soaring demand for this radiotracer. We have previously developed a good RCY (radiochemical yield) radiolabeling of [^68^Ga]Ga-FAPI-46 on an Trasis EasyOne module that satisfies the GMP requirements of clinical trials. Somehow, EasyOne can only be operated with a single germanium-68/gallium-68 generator elution. Considering the potential applications of [^68^Ga]Ga-FAPI-46, especially in a comprehensive cancer institution, we deem it appropriate to propose a large-scale production. Such a scale-up is usually achieved by combining several eluates typically coming from two-to-three different generators. This strategy comes with its own challenges, such as a larger reaction volume and greater difficulties to buffer the pH of the combined eluates. Procedures reported in the literature for automated radiolabeling of [^68^Ga]Ga-FAPI-46 did not address this need as they relied on a single generator connected to either the Eckert & Ziegler (E&Z), Trasis, or Synthra modules (Spreckelmeyer et al., 2020; Alfteimi et al., 2022; Da Pieve et al., 2022; Nader et al., 2022). Although the protocol proposed by Spreckelmeyer et al. included a prepurification step to ensure the compatibility of the process with a wide variety of generators, the addition of this prepurification step has not been considered to gather elutions from multiple generators, which is precisely the purpose of this work.

To address this unmet need for scaled-up production, we sought to re-investigate the radiolabeling of [^68^Ga]Ga-FAPI-46. Here, we propose a versatile, robust, and GMP-compliant automated process for the production of [^68^Ga]Ga-FAPI-46 on the Trasis miniAllinOne (miniAIO) module by incorporating a prepurification step compatible with multiple elutions from the most widely used germanium-68/gallium-68 generators.

## 2 Results and discussion

### 2.1 Experimental design

**FIGURE 1:**
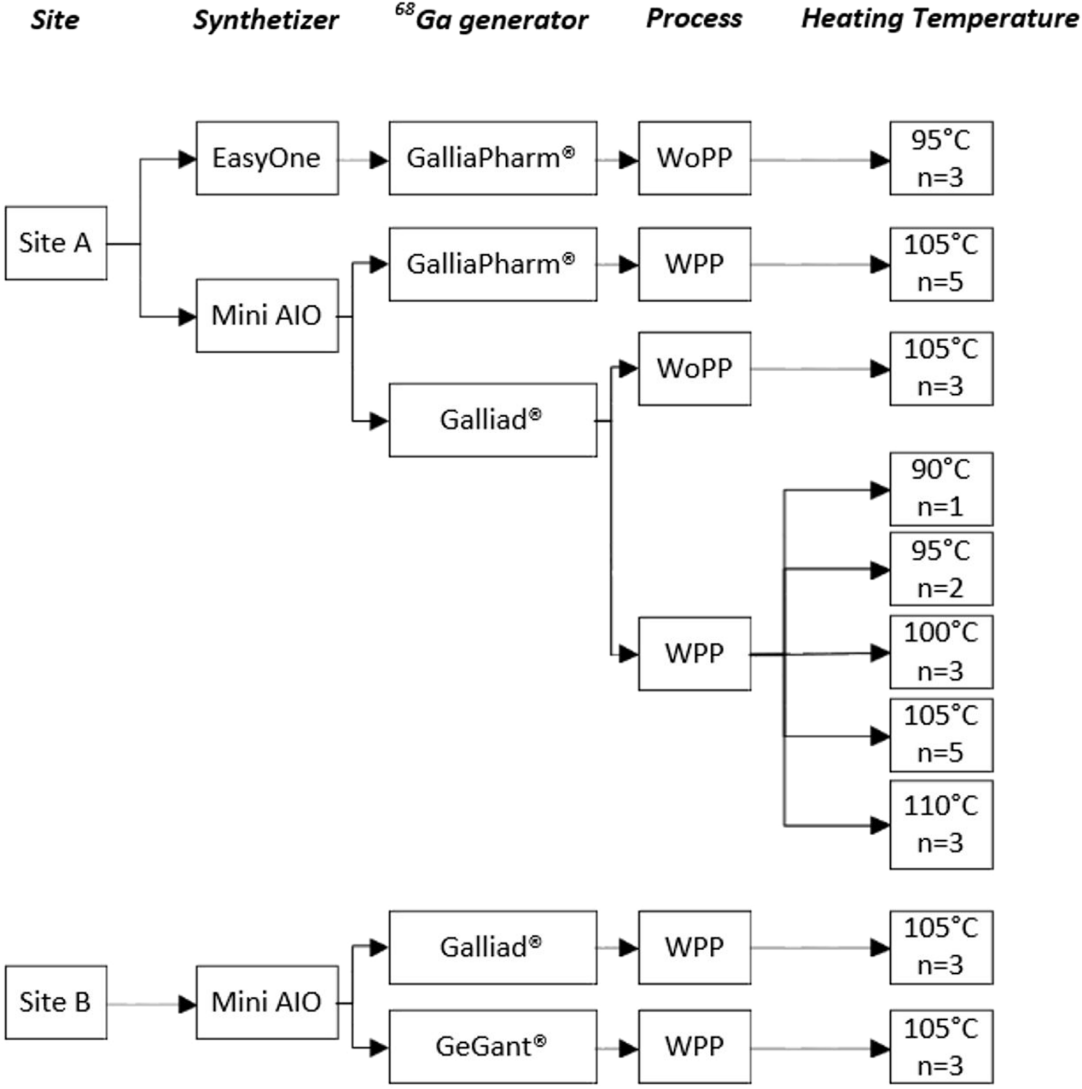
Experimental plan for the optimization of the automated [^68^Ga]Ga-FAPI-46 production process. Abbreviations: WPP: with prepurification; WoPP: without prepurification; site A Institut Curie; site B Trasis.

The optimization of the [^68^Ga]Ga-FAPI-46 production process was divided into three steps (Figure 1): i) the optimization of the gallium-68 prepurification by testing various chemistries of cationic solid-phase extraction (SPE) cartridges, ii) the adaptation of the radiolabeling step to the Trasis miniAIO synthesizer to maximize the yield and (radio)chemical purity, and iii) the verification of process robustness toward various models of generators and volumes of eluates.

To increase statistical significance and demonstrate a practical example of technology transfer, experiments were carried out in replicates on two different sites by different teams of experimenters (Figure 1, site A and B, respectively). Experimental protocols and quality control (QC) checks for each of these experiments are detailed in Section 4 ; Supporting Information S1

### 2.2 Gallium-68 prepurification

Improvements in the quality of gallium-68 generators and reduction in germanium-68 breakthrough have led to the generalization of the radiolabeling process without prepurification of the incoming activity. However, each generator has its specific volume/molarity of acidic eluent and, consequently, a dedicated volume/molarity of reaction buffer. This diversity complicates the compatibility of the production process among several brands/models of generators. Additionally, most automated processes without prepurification cannot cope with larger volumes of [^68^Ga]GaCl_3_ resulting from the combination of multiple generators’ elutions. Including a prepurification step in the present automated process was designed to overcome these limitations.

So far, two approaches have been developed to desorb [^68^Ga]GaCl_3_ from cationic SPE cartridges during the prepurification step. The first one consists in using a mixture of solvents such as acetone and HCl (de Blois et al., 2011; Šimeček et al., 2013). This mixture enables a low volume of eluent but has the disadvantage of introducing a high amount of class-3 toxic solvent into the reaction mixture and, thus, potentially in the final product. As a consequence, a quantification of the residual acetone by gas chromatography (GC) would be required for the pharmaceutical release of the product. To avoid the necessity of this quality control, we opted for the well-known second approach based on a mixture of concentrated NaCl and HCl (Mueller et al., 2012). The eluent composition was 5 M NaCl in 2.2 mL of HCl 0.5 M, which ensures a targeted pH of 4 during radiolabeling. We decided to test different ionic interaction strengths as the literature only describes the use of strong cationic exchange (SCX) cartridges. The three following SPE cartridges could be categorized as a weak cationic exchange (WCX), namely, Oasis plus short WCX, Chromafix HR-XCM (M), and Sep-Pak Light CM, and the following two can be categorized as SCX: Oasis Plus short MCX and Chromafix PS-H^+^ (S). Except the Sep-Pak Light CM, we preferred using cartridges with a copolymer support compatible with aqueous acidic mixtures at pH < 2, which is not the case for silica-based solid support. Finally, we chose SPE cartridges presented with the input/output lueur format that could be safely connected to the cassettes and tubing of the miniAIO module. The cartridges were tested under standard elution conditions to determine the most appropriate cartridge (Table 1). The Oasis Plus short MCX cartridge (Table 1, entry 1) demonstrated excellent trapping of the activity, but the recovery after elution proved difficult, with almost one-third of [^68^Ga]GaCl_3_ remaining on the cartridge. We did not select this cartridge, but it could nevertheless be of interest. Its use would, therefore, involve adaptations such as a larger volume of eluent or an increase in the NaCl salt concentration. Conversely, with WCX cartridges (Table 1, entries 2–4), interactions were not sufficient to trap more than 50% of [^68^Ga]GaCl_3_ but allow for a near-quantitative desorption of it. Notably, preconditioning of these cartridges (as described in Table 2) did not yield any improvement in trapping (data not shown). Overall, the SCX Chromafix PS-H^+^ (S) (Table 1, entry 5) demonstrated the best efficiency with quantitative trapping and >93% of recovery. This last cartridge was, therefore, selected for the rest of the study.

**TABLE 1:** Influence of the type of SPE cartridge used for the prepurification step.

Entry	Cartridge^a^	Trapping (%)	Recovery< (%)
1	Oasis plus short MCX	100.0	62.9
2	Oasis plus short WCX	50.0	98.2
3	Chromafix HR-XCW (M)	34.0	98.9
4	Sep-Pak Light CM	13.4	99.0
5	Chromafix PS-H^+^ (S)	99.9 ± 0.1^b^	93.9 ± 0.5^b^

^a^ Conditions: volume of [68Ga]GaCl_3_: 1.1 mL from GalliAd^®^.

Eluent flow rate: 2 mL/min, eluent volume: 2.2 mL, and without preconditioning or rinsing of the cartridge.

^b^ Experiment run in triplicate.

**TABLE 2:** Influence of the elution parameters on the prepurification efficiency.

Entry	SPE (Chromafix PS-H+ (S)) cartridge		Eluent		Trapping (%)	Recovery (%)
	Preconditioning	Rinsing	Flow rate (min)	Volume (mL)		
1	None	None	2 mL^−1^	2.2	99.9 ± 0.1 *^a^*	93.9 ± 0.5 *^a^*
2	1 mL EtOH +5 mL NaCl 0.9%	None	2 mL^−1^	2.2	99.9	94.1
3	1 mL EtOH +5 mL HCl 0.1 M	None	2 mL^−1^	2.2	99.8	92.4
4	1 mL EtOH +5 mL water	None	2 mL^−1^	2.2	99.9	93.8
5	5 mL NaCl 0.9%	None	2 mL^−1^	2.2	99.6	92.4
6	None	2.5 mL NaCl 0.9%	2 mL^−1^	2.2	99.9	86.2
7	None	5 mL NaCl 0.9%	2 mL^-1^	2.2	99.9	87.6
8	None	None	1 mL^−1^	2.2	99.7	92.2
9	None	None	5 mL^−1^	2.2	99.7	85.5
10	None	None	2 mL^−1^	1.25	99.8	66.1
11	None	None	2 mL^−1^	1.7	99.9	87.2
12	None	None	2 mL^−1^	2.5	99.7	94.1

^a^ Experiment run in triplicate.

We then studied the effect of four different elution parameters that we considered critical for the prepurification efficiency of a 1.1-mL [^68^Ga]GaCl_3_ eluate from the GalliAd^®^ generator (Table 2). Preconditioning of the SPE cartridge is often included in gallium-68 labeling protocols (Ocak et al., 2010; Schultz et al., 2013). Here, preconditioning tests (Table 2, entries 2–5) showed similar results as those of the initial conditions without preconditioning (entry 1). Quantitative trapping and recovery ranging from 92.4% to 94.1% were observed. Since it did not prove beneficial, and to simplify operations, preconditioning of the SPE cartridge was omitted. Next, to remove potential incoming impurities from the generator, we investigated the impact of rinsing the SPE cartridge post-trapping (de Blois et al., 2011; Šimeček et al., 2013). Rinsing with saline, with whichever volume used, proved detrimental to the recovery (<90%, entries 6 and 7). This is due to losses of the trapped activity during the rinse with a saline solution. Unfortunately, the cassette layout does not allow the introduction of another non-ionic rinsing solvent such as water for injection. Although lowering the flow rate of desorption did not affect the recovery (entry 8), increasing it to 5 mL .min^−1^ proved detrimental, with recovery falling below 86% (entry 9). Finally, we investigated the impact of the volume of the eluent mix on the recovery. Volumes below 2 mL significantly reduced the recovery (entries 10–11), while increasing the volume to 2.5 mL did not improve it (entry 12). Given the steep drop in recovery at volumes below 2 mL, we opted for a final optimized volume of eluent of 2.2 mL. Should a larger safety margin be necessary to account for the process variation, this volume could be increased to 2.5 mL. Even if trapping or recovery improvements were not perceptible, the fluid was optimized to save time and, thus, contributed to a better activity yield (AY).

To assess the suitability of the prepurification conditions with multiple generator elutions, we tested this procedure with 1 × 5 mL, 2 × 5 mL, and 3 × 5 mL of eluate (see Material and Methods). With all three volumes, the trapping efficiency was ≥99.8%, and the percentages of recovery were 92.7%, 94.1%, and 94.0%, respectively. This demonstrates the compatibility of our procedure with volumes corresponding to up to three generators in the worst-case scenario of 5 mL elutions (e.g., three GalliaPharm^®^ generators).

### 2.3 Radiolabeling optimization

**FIGURE 2:**
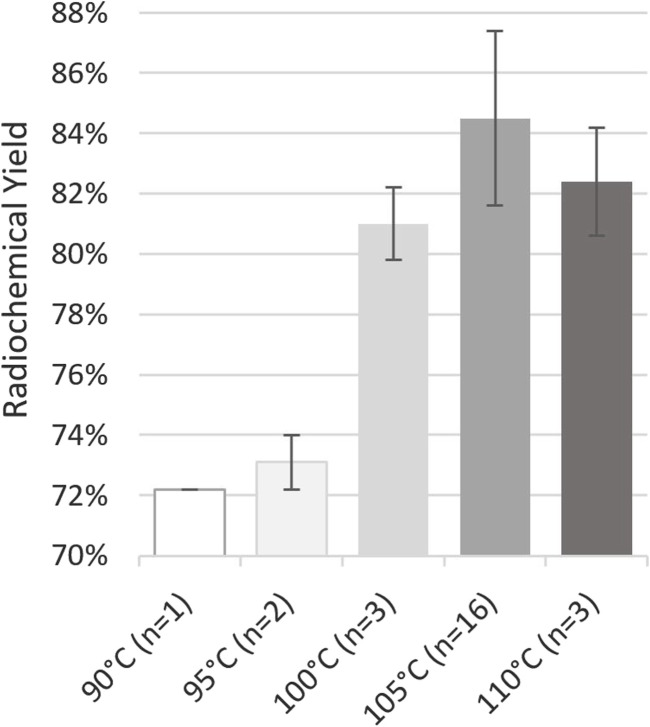
Impact of temperature on [^68^Ga]Ga-FAPI-46 radiochemical yield after 10 min of heating. These tests were performed on GalliAd^®^ on site A. Conditions: volume of [^68^Ga]GaCl_3_: 1.1 mL from GalliAd^®^, prepurification on Chromafix PS-H^+^ (S), eluent flow rate: 2 mL/min, eluent volume: 2.2 mL, without preconditioning or rinsing of the cartridge, and labeling for 10 min at the indicated temperature.

**FIGURE 3:**
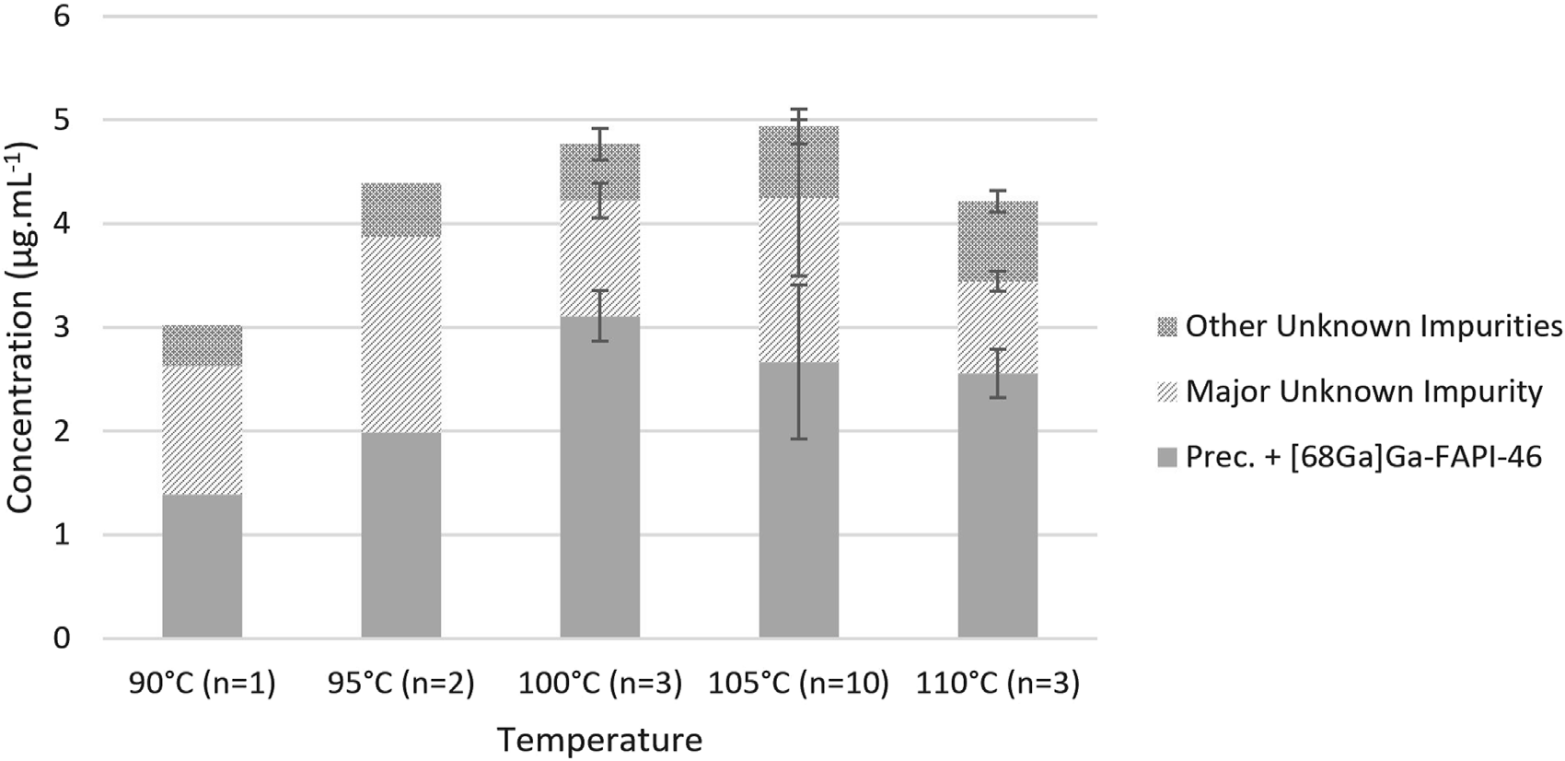
Influence of the reaction temperature on the chemical purity. Conditions: volume of [^68^Ga]GaCl_3_: 1.1 mL from GalliAd^®^, prepurification on Chromafix PS-H^+^ (S), eluent flow rate: 2 mL/min, eluent volume: 2.2 mL, without preconditioning or rinsing of the cartridge, and labeling for 10 min at the indicated temperature.

Given the minimalism of radiolabelings with gallium-68, few parameters, pH, heating temperature, and duration, may be varied. The composition of the eluent was determined to obtain a targeted pH of 4 in the reaction mixture, which is particularly suitable for the radiolabeling of the DOTA chelator (Mueller et al., 2012). Regarding heating temperature and duration, Alfteimi et al. (2022) reported that it could be reduced below 10 min at 94°C while offering a RCY ≥89%. Conversely, our previous tests conducted on EasyOne at 95°C for 5 min showed a significant decrease in both RCY, below 83%, and radiochemical purity (RCP), below 98%, while heating at 90°C for 10 min did not. Based on previously published protocols (Spreckelmeyer et al., 2020; Da Pieve et al., 2022) and our experience in the former [^68^Ga]Ga-FAPI-46 development, we decided to set the radiolabeling duration at 10 min and study the effect of the reaction temperature (Figure 2). Still, initial tests on miniAIO at 90°C–95°C yielded poor RCY, below 75%, and analysis of the remaining activity in the waste showed the presence of free [^68^Ga]GaCl_3_ that had not been chelated. Increasing the temperature improved the yield with a sudden enhancement in reactivity above 100°C. The maximum average RCY was found to be reached at 105°C, yielding [^68^Ga]Ga-FAPI-46 in 85% ± 3% (*n* = 16). It is noteworthy that the pre-heating of the reactor during the gallium-68 elution proved to be critical to achieve high RCY (data not shown). RCP of the final formulated product was found to be independent of the radiolabeling temperature. For all the temperatures tested, RCP was constantly >99% (data not shown). On the other hand, we observed small variations in the chemical purity (CP) with the reaction temperature (Figure 3). The CP was determined using an in-house UHPLC method specifically designed for the QC of [^68^Ga]Ga-FAPI-46 (see Supporting Information S1). Two main peaks were identified. The first peak corresponds to the *precursor + *[** ^*68*^ *Ga*]*Ga-FAPI-46,* two compounds that are known to be difficult to resolve (Jussing et al., 2021). The second major peak corresponds to an unknown impurity, which is designated as *major unknown impurity (impurity 2)*. The latter is hypothesized to result from the chelation with another metallic cation than gallium(III). UHPLC analysis of the FAPI-46 precursor chelated with non-radioactive zinc(II) showed similar retention time and UV spectrum as impurity 2. It is consistent with the substantial amount of zinc(II) commonly found in the [^68^Ga]GaCl_3_ eluate (Velikyan, 2015). When detected, the smaller peaks were combined as a *sum of other unknown impurities*. Overall, we observed a slight tendency to an increase in the sum of all impurities with the reaction temperature, with a maximum of 4.9 ± 0.6 μg .mL^-1^ at 105°C. However, there was no statistically significant differences in the overall CP between 100 °C, 105 °C, and 110 C °(ANOVA, α = 0.95). To the best of our knowledge, the CP was not analyzed or poorly described in previous reports of [^68^Ga]Ga-FAPI-46 labeling, making it difficult to compare. Based on, Jussing et al. (2021), who proposed a limit of overall CP ≤10 μg .mL^−1^, a specification of ≤8 μg .mL^−1^ seems consistent with our analyses. In summary, we found that labeling of [^68^Ga]Ga-FAPI-46 on Trasis miniAllinOne was best carried out at 105°C for 10 min with the inclusion of a reactor and precursor pre-heating during the elution/prepurification step.

### 2.4 Robustness toward various generators

Having established the prepurification and labeling conditions, we sought to test this novel protocol on various models of generators (Table 3). With regards to the RCY or AY, no statistical differences were observed using one of the three tested generators (ANOVA, α = 0.95). The QC of the final products (RCP and CP) was identical with those of two/three tested generators, assuming that CP should be equivalent with the GeGant^®^ generator. Therefore, these results clearly demonstrate the compatibility of the present optimized process with several models of generators.

**TABLE 3:** Testing the robustness of the optimized process with various generators.

	GalliAd^®^ (*n* = 8)	GalliaPharm^®^ (*n* = 5)	GeGant^®^ (*n* = 3)
Starting activity (MBq)	477 ± 221	496 ± 27	202 ± 27
Activity yield (%)	74.1 ± 5.5	76.0 ± 0.8	74.1 ± 0.7
Radiochemical yield (%)	88.4 ± 3.3	91.0 ± 0.4	90.3 ± 0.8
Molar activity (GBq.µmol^-1^)	11.6 ± 3.4*	7.2 ± 0.5	n.d
Radiochemical purity r-UHPLC (%) [^68^Ga]Ga-FAPI-46 Other [^68^Ga]Ga-impurities	99.6 ± 0.2 0.4 ± 0.2	99.7 ± 0.2 0.3 ± 0.2	99.5 ± 0.3 0.5 ± 0.3
Radiochemical purity r-TLC(%) [^68^Ga]Ga-FAPI-46 [^68^Ga]Ga(III)-chloride [^68^Ga]Ga-colloids	99.6 ± 0.1 0.2 ± 0.2 0.3 ± 0.1	99.5 ± 0.1 0.2 ± 0.1 0.3 ± 0.1	99.8 ± 0.1 0.1 0.2 ± 0.1
Chemical purity (µg.mL^-1^) Prec. + [^68^Ga]Ga-FAPI-46 Major unknown impurity Other impurities	2.7 ± 1.5* 1.4 ± 0.7* 0.6 ± 0.3*	2.6 ± 0.9 1.7 ± 1.1 0.8 ± 0.1	n.d

Results given as average ±standard deviation; *n = 3; n.d., not determined.

### 2.5 Process validation and comparison

To demonstrate practical applicability of the developed methodology, we ran a production of [^68^Ga]Ga-FAPI-46 using the optimized process with prepurification on Trasis miniAllinOne with single or three different elutions and compared them to other previously established protocols without prepurification on miniAllinOne and EasyOne (Table 4). Experiments were carried out under operating conditions mimicking those of routine clinical productions. Specifications listed in Table 4 were chosen based on European Pharmacopoeia, ICH Q3 guidelines, and the known literature for [^68^Ga]Ga-FAPI-46, in particular, the CP specification (<8.0 μg mL^−1^) and the results of the present study.

**TABLE 4:** Comparison of the optimized single and multi-elution process with prepurification and two other established protocols.

	Specification	EasyOne WoPP Single elution (*n* = 3)	miniAIO WPP Single elution (*n* = 16)	miniAIO WoPP Single elution (*n* = 3)	miniAIO WPP Multi-elution (n = 3)
Starting activity (MBq)	n.a	981 ± 24	431 ± 190	569 ± 83	1755 ± 92
Activity yield (%)		n.r	73.5 ± 4.2	77.3 ± 1.2	57.3 ± 1.3
Radiochemical yield (%)		n.r	89.5 ± 2.6	91.9 ± 1.5	89.4 ± 0.4
Duration (min)		19.0	23.6 ± 1.1	23.7 ± 1.5	39.0
Radiochemical purity r-UHPLC (%) [^68^Ga]Ga-FAPI-46 Other [^68^Ga]Ga-impurities	≥95.0% ≤5.0%	99.3 ± 0.2 0.7 ± 0.2	99.6 ± 0.2 0.4 ± 0.2	99.2* 0.8*	99.2 ± 0.1 0.8 ± 0.1
Radiochemical purity r-TLC (%) [^68^Ga]Ga-FAPI-46 [^68^Ga]Ga(III)-chloride [^68^Ga]Ga-colloids	≥95% ≤2.0% ≤3.0%	98.2 ± 0.5 0.5 ± 0.3 1.3 ± 0.3	99.6 ± 0.1 0.1 ± 0.1 0.3 ± 0.1	99.5 ± 0.1 0.3 ± 0.2 0.2 ± 0.2	99.7 0.1 0.2
Chemical purity (µg.mL^-1^) Prec. + [^68^Ga]Ga-FAPI-46 Major unknown impurity Other impurities	Sum of impurities <8.0 μg.mL^-1^	3.5 ± 0.2 1.4 ± 0.2 1.9	2.7 ± 0.7** 1.6 ± 0.8** 0.7 ± 0.2**	2.9* 1.1* 0.4*	2.6 ± 0.3 3.0 ± 0.3 0.8 ± 0.2
pH	4.0–8.0	6.0	6.0	6.0	6.0
Ascorbate concentration (mg.L^-1^)	≥300	≥300	≥300	≥300	≥300
Sterile filter integrity test (automatic leak test)	Pass (≤500 μL.min^-1^)	Pass	Pass	Pass	Pass
Ethanol % v/v	<10%	7.1 ± 0.2	7.9 ± 0.8	8.7 ± 0.3	n.d

Results given as average ±standard deviation; n.a, not applicable; n.d, not determined; n.r, not reported; WPP, with prepurification; WoPP: without prepurification; * *n* = 1; ** *n* = 10.

RCY and AY from the previously developed EasyOne process were not reported as activities measurements could not be carried out under the conditions described in Materials and Methods. In addition, the ethanol content could not be determined in the case of high-activity radiosynthesis.

On the miniAIO, RCY or AY obtained with the single elution prepurification process was identical to the one obtained without prepurification and was comparable to those described by Alfteimi et al. (2022) using three different modules. Reproducibility proved exceptional with very little variations between the production runs, experimenters, and sites; this reliable knowledge of the process performance is pivotal for operational planning. Both the RCP and CP profiles proved almost identical in all three single elution processes, with only a marginally higher level of [^68^Ga]Ga-colloids and CP in the EasyOne process, although well within specifications. Similarly, the pH, ascorbate, and ethanol content were identical. The prepurification process developed in this study performed identically to the existing processes without prepurification.

Overall, high-activity radiolabeling, starting with 1755 ± 92 MBq of [^68^Ga]GaCl_3_ from three generators, was successfully conducted. RCY was >89% and, thus, similar to that of previous single-elution tests. Due to the short half-life of gallium-68, elution of the weakest generator first and the strongest generator last helped minimize the loss of total starting activity; however, AY was way lower, <57%. This was due to the time needed for transferring the 10-mL elution vial of [^68^Ga]GaCl_3_ from one laboratory to another and for withdrawing and loading the volume onto the SCX cartridge. As a consequence, the total radiosynthesis time increased to 39 min. Locating the generators in the same shielded box would optimize this synthesis time to 30 min in the case of triple/quadruple elution process since the miniAIO modules are equipped with double syringe drivers, enabling two elutions to be performed synchronously. We, therefore, assume that the AY should be approximately 70% for a triple/quadruple elution process. The RCP and CP profiles were found to be almost identical to those of the miniAIO prepurification processes. The only difference observed, although not significant, was the amount of *impurity 2* (ANOVA, α = 0.95). This nearly twofold increase may be attributed to the greater amount of zinc(II) content provided by the three different elutions. However, the specifications remain compliant, assuming that the ethanol content should be comparable to the single-elution prepurification process.

## 3 Conclusion

[^68^Ga]Ga-FAPI-46 is a highly promising PET imaging tracer for many types of cancer and inflammatory pathologies. Current radiolabeling processes do not allow for its large-scale production, in particular, through multiple generator elutions. We addressed this unmet need by developing a [^68^Ga]Ga-FAPI-46 production process on Trasis miniAllinOne including a prepurification step and optimized radiolabeling conditions. The robustness of the process was demonstrated with three different brands of generators. High-activity radiolabeling with up to three different elutions was successfully conducted with good RCY (>89%, *n* = 3). The QC complies with internal specifications based on European Pharmacopoeia, RCP was excellent, above >99%, and CP meets the micro-dosing criteria (i.e., < 100 µg per injection) even if the entire volume of the final product is injected into a single patient. Finally, this process is suitable for GMP production and allows scaling-up of routine productions, higher throughput, and, ultimately, better patient care.

## 4 Materials and methods

### 4.1 Materials

Sep-Pak Light CM, Oasis Plus short MCX, and Oasis plus short WCX cartridges were supplied by Waters (Atlanta, GA/United States); Chromafix HR-XCW (M) and Chromafix PS-H+ (S) were supplied by Macherey-Nagel (Düren, Germany).

All cassettes and generic gallium-68 reagent kits for EasyOne and miniAllinOne were provided by Trasis (Ans, Belgium). Sodium ascorbate was supplied by Cooper (Melun, France) or Spectrum Pharmaceuticals (Boston, MA/United States). Water TraceSELECT was supplied by Honeywell (Charlotte, VA/United States). The FAPI-46 precursor was provided by SOFIE iTheranostics (Dulles, VA/United States) either as technical grade (1.0 mg vials) or GMP-grade (50-µg single-use vials). When the technical grade was used, the entire vial was dissolved in TraceSELECT water (1.0 mg/mL stock solution), and the solution (50 µL) was aliquoted in separate single-use vials to achieve the correct amount of precursor needed for one synthesis.

Gallium-68 was obtained from ^68^Ge/^68^Ga generators: GalliaPharm^®^(E&Z, Berlin, Germany) 1,850 MBq in 5 mL of 0.1 M HCl, GalliAd^®^ (IRE-Elit, Fleurus, Belgium) 1,850 MBq in 1.1 mL of 0.1 M HCl, or GeGant^®^ (Isotope Technologies Munich, Munich, Germany) 1,000 MBq in 4 mL of 0.05 M HCl.

The radiosynthesis of [^68^Ga]Ga-FAPI-46 was performed on the Trasis EasyOne and miniAllinOne synthesizer operated by the software program Supervision. The generic Gallium-68 labeling cassettes and reagent kits from Trasis were used as the base for this study; any alterations of the generic kits are explicitly described below, whenever applicable.

### 4.2 Radiochemical yield calculations

The radiochemical yield of a full radiosynthesis was calculated by dividing the final product activity measured at the end of synthesis (EoS) by the initial activity at the start of synthesis. The latter is difficult to measure in a gallium-68 process and was, therefore, back-calculated from the sum of the isolated activity in a final product vial (FPV) plus the activity measured in the recovery (waste) vial and the activity measured on all parts of the cassette, which was decay-corrected at the start of synthesis (SoS) (Eq. 1).

$$RCY=\frac{{\left(Activity\;in\;FPV\right)}_{EoS}}{{\left(Activity\;in\;FPV+Activity\;in\;waste+Activity\;on\;cassette\right)}_{SoS}}.$$ (1)

To minimize measurement approximations regarding prepurification optimization, the initial activity was eluted into a vial and measured in a dosimeter before the tests were carried out. For all other tests, the initial activity was measured in the reactor by the module’s calibrated detector after the elution(s). At the end of the test, when applicable, the final product vial and residual activity from the following parts of the cassette were measured in the dosimeter: initial elution vial, reactor, waste vial, and cationic and HLB formulation cartridges.

When applicable, the decay-corrected RCY was calculated by decay correction of the RCY to the start of synthesis.

### 4.3 Radiosynthesis of [^68^Ga]Ga-FAPI-46 without the prepurification step

#### 4.3.1 On Trasis EasyOne

*Reagent kit and cassette preparation:* the FAPI-46 precursor (50 µg) was manually solubilized by sequentially adding the following into the precursor vial: i) an aqueous solution of sodium ascorbate (10 mg. mL^-1^, 250 µL) and ii) the acetate buffer from the generic Trasis gallium-68 reagent kit. After homogenization, the resulting precursor solution was manually transferred into the reactor of the generic ^68^Ga-labeling Trasis’ cassette. To manually prepare the final product formulation, an aqueous solution of sodium ascorbate (10 mg. mL^-1^, 500 µL) was added to the final product vial.

*Process:* [^68^Ga]GaCl_3_ was eluted from the generator with 5 mL of 0.1 M aqueous HCl into the reactor containing the precursor solution preheated at 95°C. Radiolabeling was subsequently carried out at 95°C for 10 min. The crude reaction mixture was then passed through an Oasis HLB cartridge, which was then washed with saline. Purified [^68^Ga]Ga-FAPI-46 was subsequently released from the HLB with 0.9 mL of ethanol; the eluted fraction was collected in the final product vial through a 0.22-µm filter and diluted up to 10 mL with saline.

#### 4.3.2 On Trasis miniAllinOne

*Reagent kit and cassette preparation:* the FAPI-46 precursor (50 µg) was manually solubilized by adding the acetate buffer from the generic Trasis gallium-68 reagent kit to the precursor vial. After homogenization, the resulting precursor vial was placed on the cassette. Sodium ascorbate powder (540 mg) was manually added to the saline bag from the reagent kit (50 mL) using a syringe and a Luer-Lock spike; the resulting sodium ascorbate in the saline solution was thoroughly shaken before the bag was connected to the cassette.

*Process:* the content of the precursor vial and sodium ascorbate in saline (500 µL) were automatically added to the reactor at the start of the process, and the resulting solution was pre-heated at 95°C. [^68^Ga]GaCl_3_ was eluted from the generator with 1.1 mL of 0.1 M aqueous HCl into the reactor. Radiolabeling was subsequently carried out at 95°C for 10 min. The crude reaction mixture was then passed through an Oasis HLB cartridge, which was then washed with saline. Purified [^68^Ga]Ga-FAPI-46 was subsequently released from the HLB with 0.9 mL of ethanol; the eluted fraction was collected in the final product vial through a 0.22-µm filter and diluted up to 10 mL with sodium ascorbate in saline.

### 4.4 Radiosynthesis of [^68^Ga]Ga-FAPI-46 with the prepurification step on miniAllinOne

#### 4.4.1 Experimental plan for process optimization

*Prepurification optimization:* the optimization of the [^68^Ga]GaCl_3_ step was carried out by trapping a known amount of raw gallium-68 eluted from the generator on a cation exchange SPE cartridge. Subsequently, the cartridge was washed and dried (optional), and the activity was released with the eluent. The experimental parameters that varied in this optimization were the type of cationic exchange cartridge (PS-H^+^ (S), Chromafix HR-XCW (M), Oasis Plus short MCX, Oasis plus short WCX and Sep-Pak Light CM, preconditioning of the cartridge, rinsing of the cartridge after trapping, and the volume and flowrate of the eluent used for the release. Multiple generator elutions were replicated by manually preparing large volumes of the eluate and diluting [^68^Ga]GaCl_3_ obtained from GalliAd^®^ up to 15 mL with 0.1 M HCl; this upper-limit was chosen from the maximum volume obtained from three elutions of the GalliaPharm^®^ generator.

*Radiolabeling optimization:* the FAPI-46 precursor (50 µg) was manually solubilized by sequentially adding the following to the precursor vial: i) an aqueous solution of sodium ascorbate (10 mg. mL^-1^, 250 µL) and ii) the acetate buffer from the generic Trasis gallium-68 reagent kit. After homogenization, the resulting precursor vial was placed for Trasis ^68^Ga-labeling *with a prepurification* cassette. To manually prepare the final product formulation, an aqueous solution of sodium ascorbate (10 mg .mL^-1^, 500 µL) was added to the final product vial. [^68^Ga]GaCl_3_ was purified as optimized in the previous step and transferred into the reactor containing the precursor solution preheated at a given temperature (range 90°C–110 °C). Radiolabeling was subsequently carried out at that temperature for 10 min. The crude reaction mixture was then passed through an Oasis HLB cartridge, which was then washed with saline. Purified [^68^Ga]Ga-FAPI-46 was subsequently released from the HLB with 0.9 mL of ethanol; the eluted fraction was collected in the final product vial through a 0.22-µm filter and diluted up to 10 mL with saline. Three main criteria were monitored for this optimization: the radiochemical yield (the higher the better, target >80%), the radiochemical purity (the higher the better, target >93%), and the chemical impurity content (the lower the better, target <8 μg. mL^-1^).

#### 4.4.2 Final optimized process

Reagent Kit & Cassette Preparation: The FAPI-46 precursor (50 μg) was manually solubilized by adding into the precursor vial the acetate buffer from the generic Trasis Gallium-68 reagent kit. After homogenization, the resulting precursor vial was placed on the cassette. Sodium ascorbate powder (540 mg) was manually added into the saline bag from the reagent kit (50 mL) using a syringe and a Luer-Lock spike; the resulting sodium ascorbate in saline solution was thoroughly shaken before the bag was connected to the cassette.

*Single elution process:* the content of the precursor vial and sodium ascorbate in saline (500 µL) were automatically transferred into the reactor at the start of the process, and the resulting solution was pre-heated at 105°C. [^68^Ga]GaCl_3_ was eluted from the generator(s) according to manufacturer’s specifications, and the resulting solution was passed through a Chromafix PS-H+ (S) SPE cartridge; if several generators were used, the process was repeated (up to three times in total). Chromafix PS-H+ (S) was then flushed with air (10 mL) before the activity was released into the reactor with the eluent (2.2 mL, 2 mL.min^−1^). Radiolabeling was subsequently carried out at 105 °C for 10 min. At the end of labeling, the crude reaction mixture was diluted into sodium ascorbate in saline (4 mL), and the resulting solution was passed through an Oasis HLB cartridge, which was then washed with sodium ascorbate in saline (2 × 4 mL). Purified [^68^Ga]Ga-FAPI-46 was subsequently released from the HLB with 0.9 mL of ethanol; the eluted fraction was collected in the final product vial through a 0.22-µm filter and diluted up to 10 mL with sodium ascorbate in saline.

*Multi elution process:* [^68^Ga]GaCl3 was obtained from an elution of an IRE generator and a vial containing 10 mL of [^68^Ga]GaCl3 from two elutions of two different E&Z generators located in another laboratory. The amount of 10 mL was automatically withdrawn from the vial and trapped on the SCX cartridge after the elution of the IRE generator, and then, the process was identical to the single elution one.

### 4.5 Quality controls

pH: pH was determined using a pH 4.0–7.0 strip (Macherey-Nagel GmbH & Co.).

Chemical and radiochemical purity: samples of the final formulated product were analyzed on an Acquity UPLC^®^ H-Class (Waters, Atlanta, GA, United states) system equipped with a UV detector set at 280 nm and a gamma detector Posi-RAM (LabLogic, Shefield, United Kingdom). The stationary phase was an Acquity HSS T3 VanGuard™ FIT; 1.8 μm; 2,1 × 50 mm (Waters), while the mobile phase was a gradient of water and acetonitrile both containing 0.1% v/v of trifluoroacetic acid. Chromatograms were analyzed using the Laura^®^ (LabLogic) and Empower^®^ (Waters) software packages. The radiochemical purity was also determined by radio-TLC using either a scan-RAM (LabLogic, Shefield, United Kingdom) controlled by Laura^®^ software (LabLogic) or on a MiniGita (Elysia, Angleur, Belgium) controlled by GINA Star TLC™ software (Elysia). Two r-TLC methods were employed. *Method A*, for the quantification of free [^68^Ga]Ga(III) chloride: the sample was spotted on a silicate gel 60 F254 (Merck) plate and eluted in 0.1 M aqueous sodium citrate. *Method B*, for the quantification of [^68^Ga]Ga colloids: the sample was spotted on an iTLC silicate gel (Agilent) paper and eluted in a 50:50 v/v mixture of 1 M sodium acetate in water and methanol.

Residual solvents: ethanol percentage in the final product was determined by gas chromatography (GC model 7890 Agilent) with a flame ionization detector.

Sodium ascorbate content: the sodium ascorbate concentration was semi-quantitatively determined with the MQuant™ strip (Merck).

Sterile filter integrity: the integrity of the sterile filter was automatically verified by the synthesizers at the end of the production process according to validated Trasis procedures.

More information on the QC methods can be found in the Supplementary Material.

## Data Availability

The original contributions presented in the study are included in the article/Supplementary Material; further inquiries can be directed to the corresponding author.
