# Supplementary material for: Development of a versatile [68Ga]Ga-FAPI-46 automated synthesis suitable to multi-elutions of germanium-68/gallium-68 generators
Source: Front Chem. 2024 Jul 15;12:1411312. doi: 10.3389/fchem.2024.1411312 (PMC11284080; doi:10.3389/fchem.2024.1411312)
Supplement: Supplementary file 1 [file DataSheet1.docx]

Supplementary Material


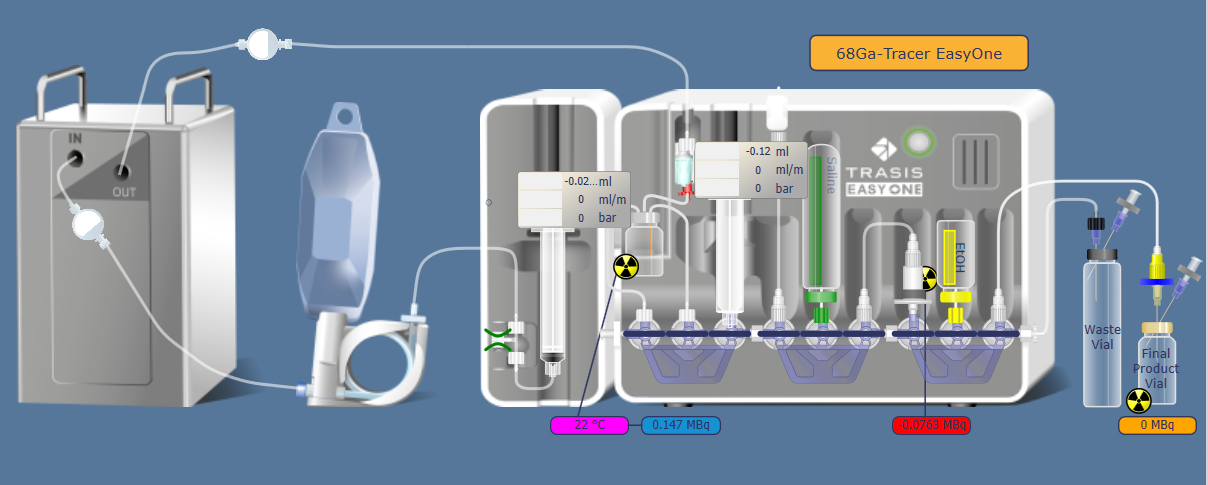


Supplementary Figure 1 Lay-out of [^68^Ga]Ga-FAPI-46 radiosynthesis on EasyOne module (Trasis) equipped with EasyOne cassette (Trasis) and reagents from Trasis reagent kit including; green vial : 30 mL of saline, yellow vial : 0.9 mL of ethanol; HLB cartridge (Waters); 0.2 µm sterile filter (Supor® polyethersulfone membrane, PharmAssure, PALL); 15 mL final product sterile vial (Fill-Ease).

Before running the sequence, the 50 µg FAPI-46 precursor vial is first taken up by a syringe of acetate buffer (Trasis) and 0.25 mL of sodium ascorbate at 10 mg/mL (Cooper), then transferred to the reactor. In final product vial, 0.5 mL of the same sodium ascorbate solution is added.


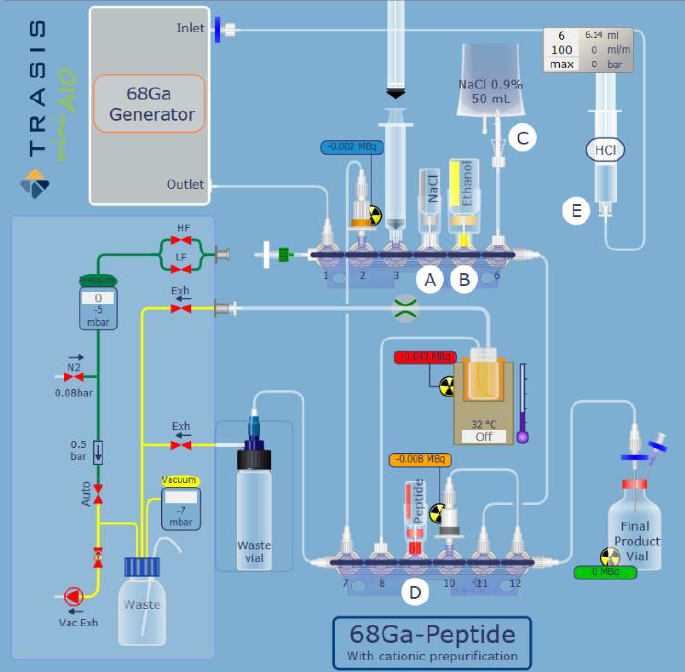


Supplementary Figure 2 Lay-out of [^68^Ga]Ga-FAPI-46 radiosynthesis with prepurification on mini-AIO module (Trasis) equipped with Trasis manifolds. Reagents; orange cartridge : Chromafix PS-H+ (S) (Macherey-Nagel) ; A : eluent solvent for cationic cartridge (Trasis); B : 5 mL ethanol vial (Trasis); C : 50 mL saline bag (B.Braun); D : 50 µg FAPI-46 precursor vial (Sofie) + sodium acetate buffer (Trasis); E : 5 mL HCl 0.1M syringe (Trasis); grey cartridge : HLB Oasis (Waters); 0.2 µm sterile filter (Supor® polyethersulfone membrane, PharmAssure, PALL), 15 mL final product sterile vial (Fill-Ease).

Before running the sequence, the 50 µg FAPI-46 precursor vial is first taken up by a syringe of sodium acetate buffer (Trasis) before being placed on the cassette. 540 mg of sodium ascorbate (Spectrum) are manually added to the saline bag thanks to a syringe and 0.5 mL of this sodium ascorbate solution is added to the final product vial.


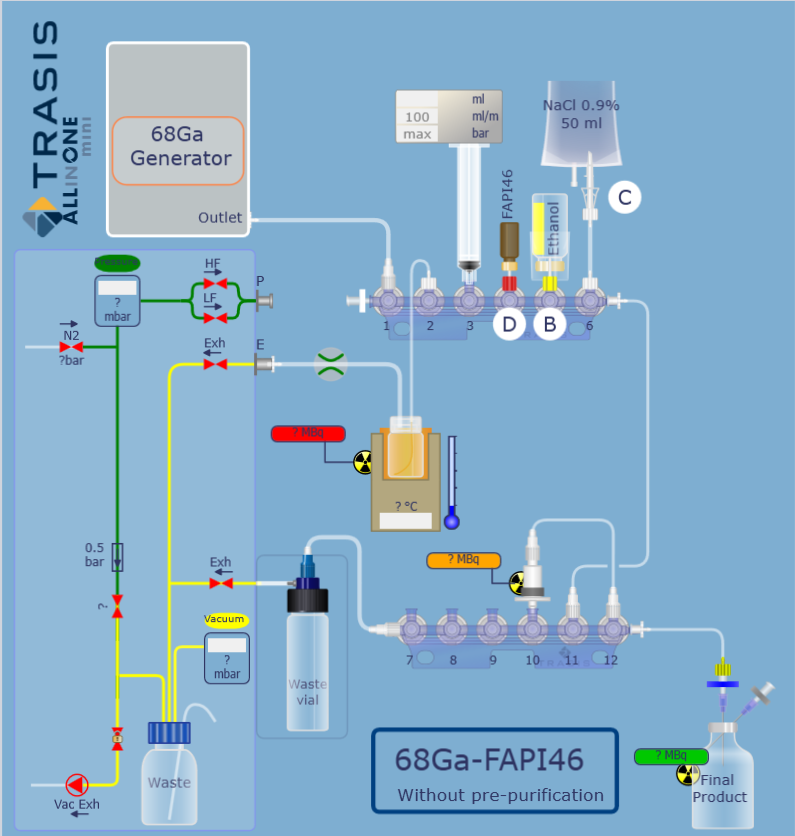


Supplementary Figure 3 Lay-out of [^68^Ga]Ga-FAPI-46 radiosynthesis without prepurification on mini-AIO module (Trasis) equipped with Trasis manifolds. Reagents; B : 5 mL ethanol vial (Trasis); C : 50 mL saline bag (B.Braun); D : 50 µg FAPI-46 precursor vial (Sofie) + sodium acetate buffer (Trasis); grey cartridge : HLB Oasis (Waters); 0.2 µm sterile filter (Supor® polyethersulfone membrane, PharmAssure, PALL), 15 mL final product sterile vial (Fill-Ease).

Before running the sequence, the 50 µg FAPI-46 precursor vial is first taken up by a syringe of sodium acetate buffer (Trasis) before being placed on the cassette. 540 mg of sodium ascorbate (Spectrum) are manually added to the saline bag thanks to a syringe and 0.5 mL of this sodium ascorbate solution is added to the final product vial.

Supplementary Table 1 : Cationic cartridges tested for prepurification step on miniAIO

| Cationic cartridges | Phase quantity | Particules size | Support | Supplier |
| --- | --- | --- | --- | --- |
| Sep-Pak Light CM | 130 mg | 37-55 µm | Silica based | Waters |
| Oasis Plus short MCX | 225 mg | 60 µm | PS/DVB | Waters |
| Oasis plus short WCX | 225 mg | 60 µm | PS/DVB | Waters |
| Chromafix HR-XCW (M) | 160 mg | 45-80 µm | PS/DVB | Macherey-Nagel |
| Chromafix PS-H+ (S) | 230 mg | 100 µm | PS/DVB | Macherey-Nagel |

PS/DVB : polystyrene-divinylbenzene

**Radio-UHPLC UV:**

Radio-UHPLC-UV analyses were performed using an Acquity UPLC H-Class® system (Waters Milford, MA, USA) coupled to a TUV detector (Waters) and a Posi-RAM γ radiodetector (Lablogic, Shefield, UK). Data acquisition and instrument control were performed using Empower® software for UV signal and Laura software for radio signal.

A gradient of (A) water + Trifluoroacetic acid (TFA) 0.1% and (B) acetonitrile + TFA 0.1%. was used at a flow rate of 0.6 mL/min on a Waters Acquity UPLC® HSS T3 VanGuard™ FIT; 1,8 µm ; 2,1 x 50 mm column. The gradient composition was under following conditions : A/B 95/5 to 88/12 in 6.5 min, 88/12 to 50/50 in 2.5 min and maintain during 1 min then return to 95/5 in 0.5 min and maintain during 2.5 min. Column temperature was maintained at 40°C. All compounds were detected at a wavelength of 280 nm. Samples were maintained at 10°C in the autosampler and injection volumes were 10 µL.

Concentration were calculated with a pre-registered calibration plot responding to y = bx + a (b : slope, a : intercept).


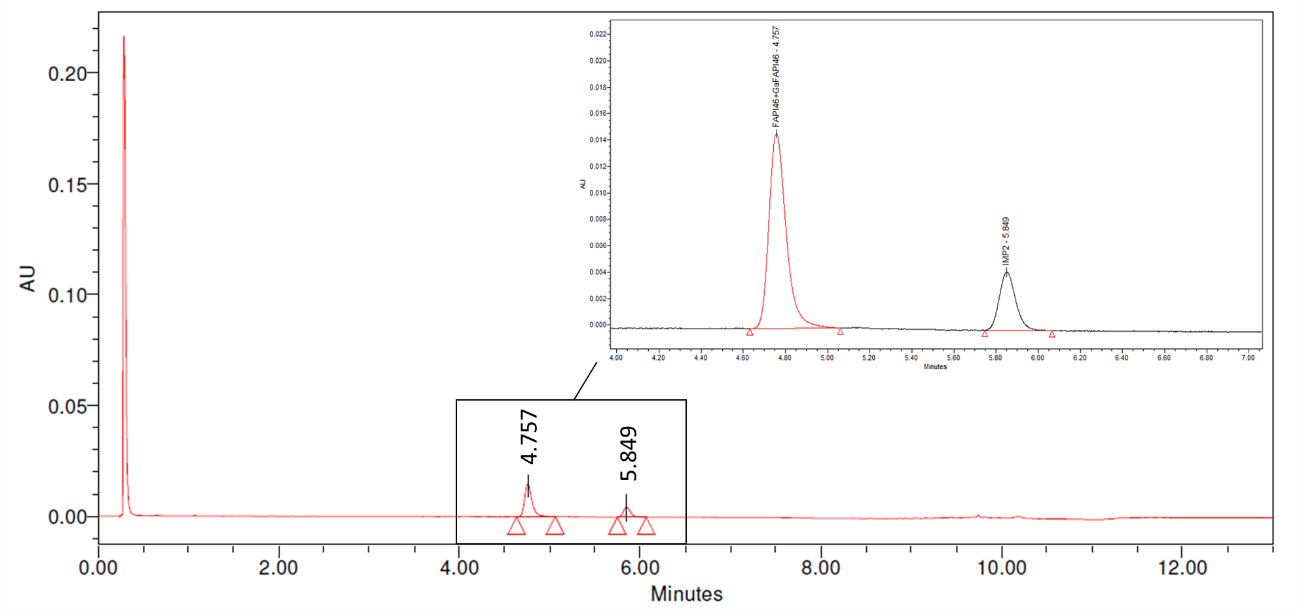


Supplementary Figure 4 Typical UV chromatogram at 280 nm of [^68^Ga]Ga-FAPI-46 final product (stationary phase : Acquity HSS T3 VanGuard™ FIT; 1,8 µm ; 2,1 x 50 mm, mobile phase : gradient of water with 0.1% TriFluoroacetic Acid (TFA) and acetonitrile with 0.1% TFA). Retention times : FAPI-46 precursor + [^68^Ga]Ga-FAPI-46 (4.76 min) ; Impurity 2 (5.85 min).


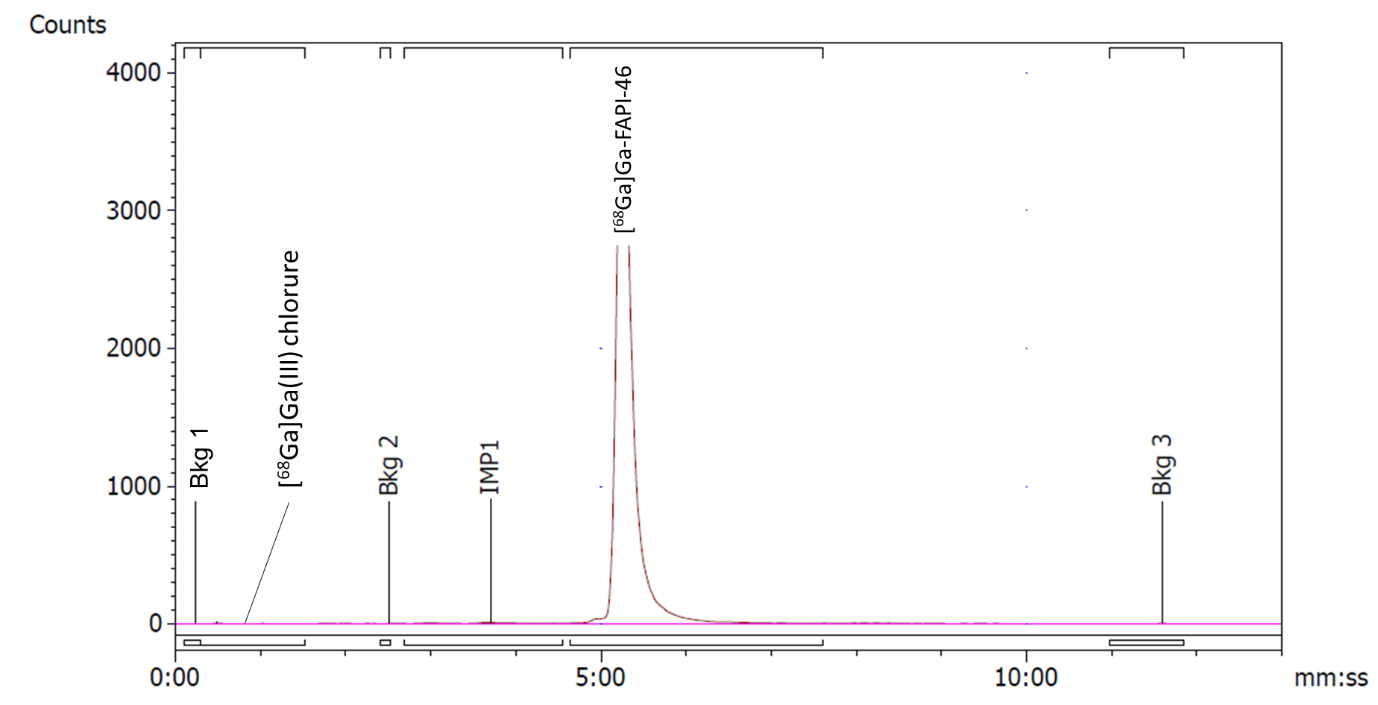


Supplementary Figure 5 Typical r-UHPLC chromatogram of final product (stationary phase: Acquity HSS T3 VanGuard™ FIT; 1,8 µm ; 2,1 x 50 mm, mobile phase : gradient of water with 0.1% TriFluoroacetic Acid (TFA) and acetonitrile with 0.1% TFA). Retention times : [^68^Ga]Ga(III) chloride (1 min 10 sec), Impurity 1 (IMP1) resulting from radiolysis (3 min 45 sec), [^68^Ga]Ga-FAPI-46 (5 min 15 sec).

**Radio-TLC**

**
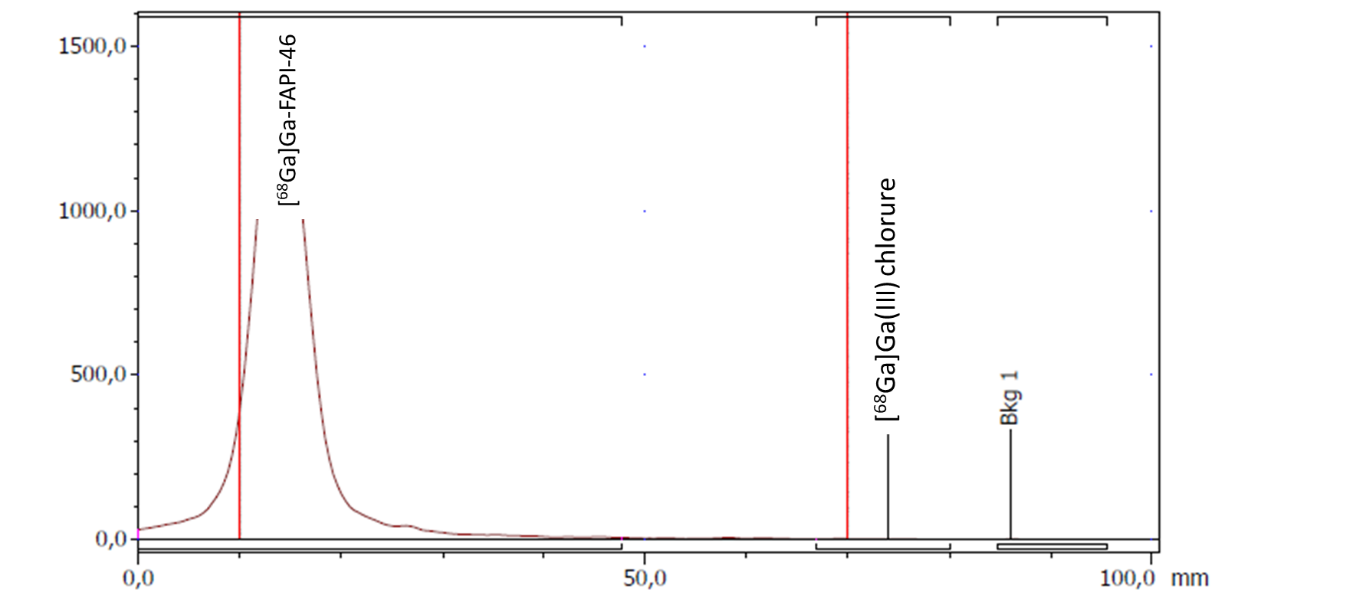
**

**Supplementary** **Figure 6** Typical r-TLC chromatogram (stationary phase : silicate gel 60 F254, mobile phase : sodium citrate 0,1 M).


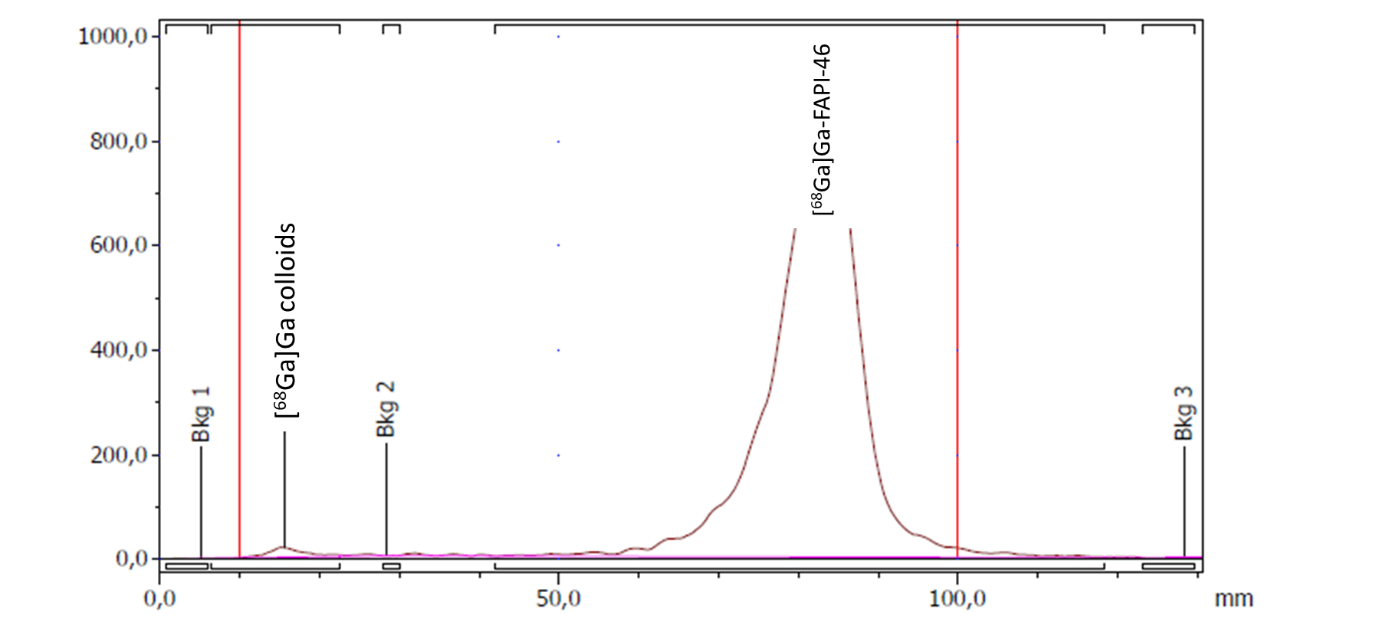


Supplementary Figure 7 Typical radio-TLC chromatogram (stationary phase: iTLC Silicate gel, mobile phase : sodium acetate 77 g/L / methanol (50/50 v:v))

**Gas Chromatography**

GC was performed on an Agilent 7890B (Agilent, Santa Clara, CA/USA) Gas Chromatograph with an autoinjector (Aglieant), a flame ionization detector (Agilent) and a J&W 14%-Cyanopropyl-phényl-méthylpolysiloxane, 30 m x 250 µm x 0,25 µm column (Agilent).

The GC was operated with Chromeleon (Thermo, Waltham, MA/USA) software under the following conditions: constant helium flow of 1.25 mL/min, oven temperature 35°C for 3.5 min, then raise to 200°C in 5.5 min, inlet temperature at 200°C. The detector temperature was 250°C, with a gas supply of 30 mL/min helium, 400 mL/min compressed air, and a nitrogen make up of 25 mL/min. A volume of 0.5 µL of samples was automatically injected with a split ratio of 25.

No calibration was plotted, but standards of 10 % ethanol (v/v) or 78.9 mg.mL^-1^ (m/v) were prepared for each day of analysis. To provide the ethanol concentration in the sample, a plot was created by forcing thru zero from the standard point value. As consequence, the equation of the plot corresponds to y = bx, (b : slope).


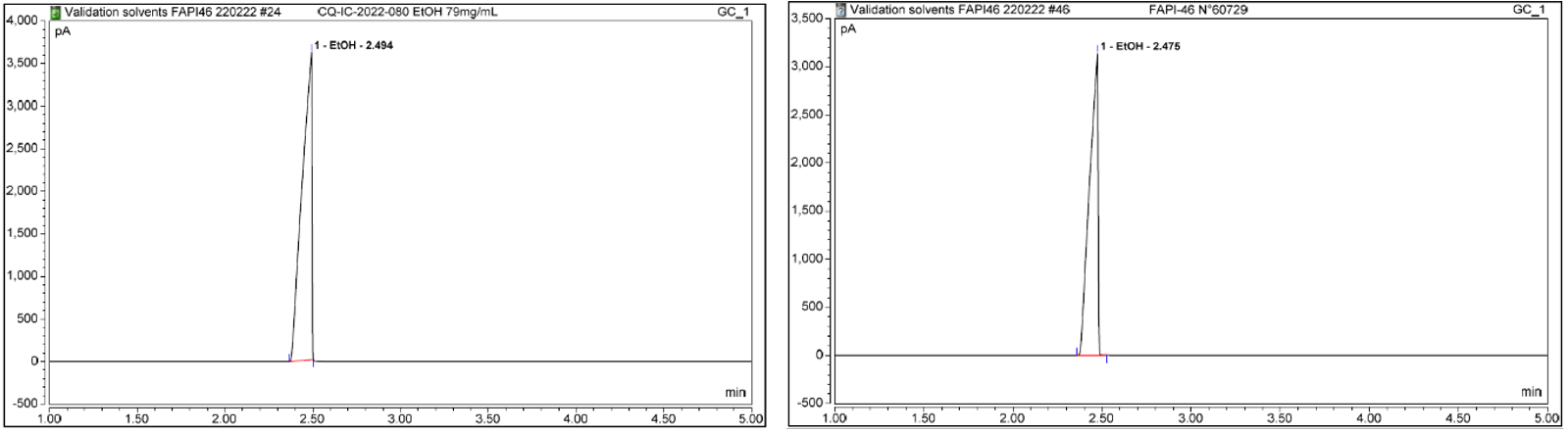


Supplementary Figure 8 Left: typical GC chromatogram of an ethanol control solution at 78.9 mg.mL^-1^ (2.49 min). Right: typical GC chromatogram of ethanol in final product sample of [^68^Ga]Ga-FAPI-46 (2.47 min).
